# Fungal microbiota in newborn infants with and without respiratory distress syndrome

**DOI:** 10.1371/journal.pone.0302027

**Published:** 2024-04-10

**Authors:** Vicente Friaza, Pilar Rojas, Carmen de la Horra, Elisa García, Rubén Morilla, Antonio Pavón, Yaxsier de Armas, Antonio J. Vallejo-Vaz, Rocío Salsoso, Francisco J. Medrano, Enrique J. Calderón

**Affiliations:** 1 Centro de Investigación Biomédica en Red de Epidemiología y Salud Pública, Madrid, Spain; 2 Instituto de Biomedicina de Sevilla, Hospital Universitario Virgen del Rocío/Consejo Superior de Investigaciones Científicas/Universidad de Sevilla, Seville, Spain; 3 Neonatology Unit, Virgen del Rocío University Children’s Hospital, Seville, Spain; 4 Departamento de Enfermería, Universidad de Sevilla, Seville, Spain; 5 Department of Clinical Microbiology Diagnostic, Hospital Center of Institute of Tropical Medicine “Pedro Kourí”, Havana, Cuba; 6 Pathology Department, Hospital Center of Institute of Tropical Medicine ʺPedro Kouríʺ, Havana, Cuba; 7 Departamento de Medicina, Hospital Universitario Virgen del Rocío, Facultad de Medicina, Universidad de Sevilla, Seville, Spain; Kobe University Graduate School of Medicine School of Medicine, JAPAN

## Abstract

**Background:**

*Pneumocytis jirovecii* infection in preterm newborns has recently been associated with neonatal respiratory distress syndrome and bronchopulmonary dysplasia. Changes in the bacterial microbiota of the airways have also been described in infants with bronchopulmonary dysplasia. However, until now there has been no information on the airway mycobiota in newborns. The purpose of this study was to describe the airway mycobiota in term and preterm newborns and its possible association with respiratory distress syndrome.

**Methods:**

Twenty-six matched preterm newborns with and without respiratory distress syndrome were studied, as well as 13 term babies. The identification of the fungal microbiota was carried out using molecular procedures in aspirated nasal samples at birth.

**Results:**

The *ascomycota* phylum was identified in 89.7% of newborns, while the *basidiomycota* phylum was found in 33.3%. *Cladosporium* was the predominant genus in both term and preterm infants 38.4% vs. 73% without statistical differences. *Candida sake* and *Pneumocystis jirovecii* were only found in preterm infants, suggesting a potential relationship with the risk of prematurity.

**Conclusions:**

This is the first report to describe the fungal microbiota of the airways in term and preterm infants with and without respiratory distress syndrome. Although no differences have been observed, the number of cases analyzed could be small to obtain conclusive results, and more studies are needed to understand the role of the fungal microbiota of the airways in neonatal respiratory pathology.

## Introduction

The World Health Organization considers as premature birth all that occurs before 37 weeks of gestation. Data based on records from 184 countries estimate preterm birth rates of 11% (5–18%) and approximately 15 million preterm births per year [1, 2]. Complications related to preterm birth cause death in the first month of 6–7 out of every 1000 newborns alive and are responsible for 14% of fatalities in children under 5 years of age, so more than a million babies die yearly due to complications of preterm birth [3].

Prematurity results in hugely changed antigen exposure. This is caused by the precipitate contact with microorganisms, nutritional antigens, and other environmental factors. In the course of pregnancy, during the last trimester, the immune system of the fetus adapts to tolerate motherly and self-antigens, whereas also making for postnatal immune defense by acquiring passive immunity from the mother. Preterm birth interrupts the influences of the intrauterine environment on the fetus that increase or decrease the risk of later disease, but also lead to premature exposure to protective or harmful extrauterine factors such as the microbiota and nutritional antigens [4].

Preterm babies are at high risk for developing respiratory diseases, mainly respiratory distress syndrome (RDS) [5]. RDS is the principal cause of morbidity and mortality in children born prematurely. The frequency and severity of RDS are inversely related to gestational age. RDS is caused by a deficiency of alveolar surfactant and an immature structure of the fetal lung [6, 7].

Recently, the complex interaction between the development of the gastrointestinal and respiratory bacterial microbiota and the regulation of immune function in newborns is beginning to be studied [8, 9]. In this line, we have described an association between RDS and primary *Pneumocystis jirovecii* infection in preterm infants [10].

However, until now, the fungal microbiome of the airways has not been studied in preterm infants, and the potential role of the mycobiota and its contribution to respiratory disease in preterm infants is unknown.

The aim of this study was to describe the fungal microbiota at birth in term and preterm infants with and without respiratory distress syndrome.

## Population and methods

A case-control study was carried out in preterm newborns (gestational age < 32 weeks and/or birth weight <1500g) admitted to the Neonatal Intensive Care Unit of the Virgen del Rocio University Hospital. We included 26 gestational age-birth and weight at birth matched infants, 13 with RDS at birth, and 13 without it. Newborns were diagnosed with RDS when they had clinical signs of respiratory distress with additional oxygen requirements and radiographic signs indicative of RDS (diffuse, fine granular opacification in both lung fields) [6]. In addition, we included 13 newborns birth at term as controls. Newborns whose mothers had HIV infection or other causes of immunosuppression were excluded.

In all cases, informed consent was obtained from the legal representatives of the newborns prior to their inclusion in the study. The study protocol was approved by the hospital’s ethics committee (protocol code 0288–N-21) and the study was developed between May 1, 2021 and April 30, 2023.

Each newborn was evaluated clinically using a standardized procedure and biological samples were collected from each one for analysis in the neonatal intensive care unit where for our protocol all premature newborns with < 32 weeks and/or birth weight <1500g are transferred from the delivery room approximately 10–15 minutes after delivery. Nasopharyngeal aspirate samples were taken in all premature newborns, just before the placement of a gastric tube used for enteral nutrition of these babies. Briefly, under sterile conditions, sterile saline was instilled by introducing a tube attached to a syringe through the nose and directed toward the nasopharynx. After a few seconds, the nasopharyngeal fluid was removed by means of an aspiration system with a commercially available suction catheter connected to the sterile recipient where secretions were collected. Samples were kept frozen at -20°C into aliquots of 250 μl until DNA was extracted, usually less than 1–2 days.

DNA was extracted using the nucleospin tissue kit (Macherey-Nagel). For digestion 5 μl of EDTA 0.5M pH 8 and 15 μl SDS 10% (w/v) were added to each sample. Proteinase K digestion at 56°C for a minimum of four hours and subsequent DNA purification were carried out according to the manufacturer’s instructions.

For fungal detection, we amplified the nuclear ribosomal internal transcribed spacer (ITS) region by seminested PCR using primers ITS-1 (5’- TCCGTAGGTGAAC-CTGCGG-3’) and ITS-4 (5’-TCCTCCGCTTATTGATATGC-3’) in the first PCR run and ITS-3 (5’- GCATCGATGAAGAACGCAGC-3’) and ITS-4 in the second PCR run. The reaction mixture of the first PCR (25μL) contain 3μl of DNA, 1X NH4 reaction buffer, 2 mM MgCl2, 200 μM of each deoxynucleotide triphosphate, 0.5 μM of each primer, and 1U of Biotaq DNApolymerase (Bioline, London, UK). It was performed under the following thermal cycling conditions: a first denaturing step at 95°C for 5 min followed by 35 cycles of 95°C for 30 sec, 55°C for 45 sec, 72°C for 30 sec and the final elongation step was extended 7 min at 72°C. The second run was carried out un-der similar conditions in a final volume of 40 μl, containing 4μl of the primary PCR reaction mixture, with 2U Biotaq DNApolymerase throughout 40 cycles. As *Pneumocystis jirovecii* is an atypical fungus with a different 18S/ITS1/5.8S/ITS2/28S complex and only one copy of the ITS2 locus in its genome, a specific nested PCR protocol was performed for the gene that encodes mitochondrial large-subunit ribosomal RNA (mtLSU rRNA) [11–13]. For the first round of amplification we used the external primers pAZ102-E and pAZ102-H that yielded a 346-base pair (bp) fragment. For the second round of amplification, we used the internal primers pAZ102-X and pAZ102-Y that produced a 260-bp product. Both rounds of PCR comprised 35 amplification cycles.

All PCRs were conducted on a T1 Thermocycler (Biometra, Göttingen, Germany). To avoid false positive reactions caused by contamination, filter-filled pipettes were used for all stages of the detection procedure. DNA extraction, preparation of the reaction mixture, PCR amplification, and detection were performed in separate rooms. A positive control was included in each reaction and all PCRs included molecular grade water as a negative control to detect any possible cross-contamination.

Amplicons were analyzed by electrophoresis on a 2% (w/v) agarose gel containing RedSafe nucleic acid staining solution and the bands were visualized by ultraviolet light. For fungal identification, the ITS2 region was directly sequenced when it was possible to slice the bands after repeating electrophoretically all the PCR volume and when it was not possible to clone the PCR products using the pGEM-T Easy Vector System (Promega, Madison, Wisconsin) in JM109 High Efficiency Competent Cells and 5 clones of every PCR selected on plates LB / ampicillin / IPTG / X-Gal were purified with NucleoSpin Plasmid (Macherey-Nagel) and sequenced. For genotype mtLSU rRNA *Pneumocystis* gene, positive PCR products were purified using Sephacryl S-400 columns (Amersham Pharmacia Biotech, Upsala, Sweden) and direct sequencing to detect polymorphisms at nucleotide positions 85 and 248.

All sequencing was performed with the BigDye Terminator v3.1 cycle sequencer kit following the standard protocol, purified by precipitation with EDTA / ethanol to remove excess dye terminators and resuspended in 12 μl of Hi-Di Formamide (Thermo Fisher Scientific) and performed in an Applied Biosystems AB3500 genetic analyzer. The quality control sequence was tested with SeqScanner_2 software (Thermo Fisher Scientific) and analyzed using MEGA7: Molecular Evolutionary Genetics Analysis version 7.0 for bigger datasets. For genotype characterization at the *P*. *jirovecii* mtLSU rRNA gene a reference sequence was utilized and for fungal identification the ITS2 sequence was compared using BLASTn with those stored in the GenBank nucleotide database and, when necessary, it was confirmed in the UNITE community unified base for DNA of fungal species: full UNITE + INSD database. Previously unknown sequences generated in this study have been deposited in the GenBank public repository database. ID: PP297122, PP297123, PP297124, PP297125, PP297126, PP297127.

### Statistical analyses

Categorial variables were shown as the number of cases with frequencies and compared applying the X2 or Fisher exact test when the data set was small and the expected frequency of any cell of the contingency table was below 5. Continuous variables were showed as medians with ranges and compared using Student’s t test or the Mann-Whitney U test for normal and nonnormally distributed variables, respectively. Spearman’s correlation analysis was used to describe the correlation between quantitative variables without normal distribution. Statistics were performed using IBM SPSS version 26.0 software (IBM Corp, Armont, New York). A *p* value of < .05 was considered significant in all cases.

## Results

The clinical characteristics of 39 newborns included in the study are summarized in Table 1.

**Table 1:** Clinical characteristics of newborns and their mothers.

Characteristics		Infants with RDS	Infants without RDS	*p-value*	Preterm newborns	Newborns a term	*p-value*
**Mothers**		No. = 13	No. = 13		No. = 26	No. = 13	
	Age years, median (IQR)	32.7 (38–31.2)	33.8 (36.5–28.5)	0.169*	33.3 (37.5–31)	29.6 (33.5–27)	0.097*
	Nulliparous	4 (30.7%)	9 (69.2%)	0.116^¶¶^	13 (50%)	5 (38.4%)	0.733^¶^
	Antibiotics in labor	8 (61.5%)	6 (46.1%)	0.431^¶^	14 (53.8%)	1 (7.6%)	0.014^¶¶^
	Chorioamnionitis	4 (30.7%)	4 (30.7%)	1^¶¶^	8 (30.7%)	1 (7,6%)	0.226^¶¶^
	Cesarean delivery	7 (53.4%)	7 (53.4%)	1^¶¶^	14(53.8%)	0 (0%)	0.001^¶¶^
**Newborns**							
	Gestational age birth, weeks, median (IQR)	29.2 (31.5–28.5)	30 (31.5–29)	0.357*	29.6 31.2–28.7)	39.5 (40–39)	<0.001**
	Birth weight, g, mean (SD)	1305.8 (257.9)	1366.9 (124.2)	0.099*	1336.3 (200.8)	3514,1 (430.6)	<0.0001**
	Male sex	7 (53.4%)	7 (53.4%)	1^¶^	14 (53.8%)	9 (69.2%)	0.495^¶¶^
	Intubated at birth	12 (92.3%)	3 (23%)	<0.001^¶¶^	15 (57.6%)	0 (0%)	<0.001^¶¶^

*Student’s t test

**Mann-Whitney U test

^¶^Chi-square test

^¶¶^ Fisher’s exact test

Among babies without RDS, it was more common that their mothers did not have previous delivery, but these did not reach statistical significance. Predictably, the need for intubation at birth was statistically significantly longer in newborns with RDS than in infants without RDS. None of the babies born at term needed intubation. For the rest of the characteristics, there were no significant differences between babies with and without RDS, nor between the maternal obstetric history of their mothers, including the use of prenatal corticosteroids. Naturally, gestational age and weight at birth were different with statistical significance.

There were no differences in the fungal taxa present in the airways between term and preterm babies. No differences in fungal taxa were related to intubation. There were no differences in fungal taxa between babies born by cesarean section and those born by vaginal delivery. The use of antibiotics in labor was associated with the presence of candida, but not other fungi, in the respiratory tract of newborns when we analyzed both term and premature newborns together. Candida was identified in 22.2% of babies when antibiotics were not used and in 63.6% when they were used (*Rho* = 0.14, *p* = 0.02). However, this relationship was not observed when we analyzed term and premature babies separately.

In total, we found 87 sequences that belong to 46 different fungal taxa considering term and preterm babies. Focus on preterm newborns, there were 37 identifications belonging to 21 taxa in the respiratory distress group and 29 identifications for 17 taxa in the non-distress group (Table 2).

**Table 2:** Sequences and percentage of phylum and genus grouped by newborn groups.

	RDS (n = 13)	No_RDS (n = 13)	Term (n = 13)	Preterm (n = 26)	All cases (n = 39)
**Identities: Sequences (Taxa)**	**37 (21)**	**29 (17)**	**21 (19)**	**66 (31)**	***87* (46)**
**Ascomycota**	**26/37 (70.2%)**	**19/29 (65.5%)**	**13/21 (61.9%)**	**45/66 (68.1%)**	**58/87 (66.6%)**
**Pezizomycotina**					
**Capnodiales**					
Cladosporium	13/37 (35.1%)	8/29 (27.5%)	5/21 (23.8%)	20/66 (30%)	25/87 (28.4%)
Hortaea	1/37(2.7%)			1/66 (1.5%)	1/87 (1.1%)
**Hypocreales**					
Acremonium	1/37(2.7%)			1/66 (1.5%)	1/87 (1.1%)
**Eurotiales**					
Aspergillus	1/37(2.7%)		2/21 (9.5%)	1/66 (1.5%)	3/87 (3.4%)
**Mycosphaerellale**					
Eupenidiella			1/21 (4.7%)		1/87 (1.1%)
**Chaetothyriales**					
Trichomeriaceae spp.		1/29 (3.4%)		1/66 (1.5%)	1/87 (1.1%)
**Pleosporales**					
Epicoccum			1/21 (4.7%)		1/87 (1.1%)
**Saccharomycotina**					
**Saccharomycetales**					
Candida	6/37 (16.2%)	5/29 (17.2%)	2/21 (9.5%)	11/66 (16.6%)	13/87 (14.9%)
Debaryomices	1/37(2.7%)	1/29 (3.4%)		2/66 (3%)	2/87 (2.2%)
Meyerozyma			1/21 (4.7%)		1/87 (1.1%)
Geotrichum Unclassified		1/29 (3.4%)	1/21 (4.7%)	1/66 (1.5%)	1/87 (1.1%) 1/87 (1.1%)
**Taphrinomycotina**					
**Pneumocystidales**					
P. jirovecii	4/37 (10.8%)	3/29 (10.3%)		7/66 (10.6%)	7/87 (8%)
**Basidiomycota**	**4/37 (10.8%)**	**7/29 (24.1%)**	**4/21 (19%)**	**11/66 (16.6%)**	**15/87 (17.2%)**
**Agaricomycotina**					
**Cantharellales**					
Ceratobasidium.		1/29 (3.4%)		1/66 (1.5%)	1/87 (1.1%)
**Corticiales**					
Lyomyces		1/29 (3.4%)		1/66 (1.5%)	1/87 (1.1%)
**Hymenochaetales**					
Tubulicrinis			1/21 (4.7%)		1/87 (1.1%)
**Polyporales**					
Perenniporia		1/29 (3.4%)		1/66 (1.5%)	1/87 (1.1%)
Ganoderma			1/21 (4.7%)		1/87 (1.1%)
**Tremellales**					
Bullera		1/29 (3.4%)		1/66 (1.5%)	1/87 (1.1%)
Cryptococcus	1/37 (2.7%)		1/21 (4.7%)	1/66 (1.5%)	2/87 (2.2%)
**Ustilaginomycotina**					
**Malasseziales**					
Malassezia	137 (2.7%)	2/29 (6.8%)		3/66 (4.5%)	3/87 (3.4%)
**Pucciniomycotina**					
**Sporidiobolales**					
Rhodotorula	1/37 (2.7%)		1/21 (4.7%)	1/66 (1.5%)	2/87 (2.2%)
**Wallemiomycotina**					
**Wallemiales**					
Wallemia	1/37 (2.7%)	1/29 (3.4%)		2/66 (3%)	2/87 (2.2%)
**Unknown**	**7/37 (18.9%)**	**3/29 (10.3%)**	**3/21 (14.2%)**	**10/66 (15.1%)**	**13/87 (14.9%)**
MS502	1/37 (2.7%)			1/66 (1.5%)	1/87 (1.1%)
Unknown 1	1/37 (2.7%)			1/66 (1.5%)	1/87 (1.1%)
Unknown 2	1/37 (2.7%)			1/66 (1.5%)	1/87 (1.1%)
Unknown 3	3/37 (8.1%)			3/66 (4.5%)	3/87 (3.4%)
Unknown 4		1/29 (3.4%)		1/66 (1.5%)	1/87 (1.1%)
Unknown 5	1/37 (2.7%)			1/66 (1.5%)	1/87 (1.1%)
Unknown 6		1/29 (3.4%)		1/66 (1.5%)	1/87 (1.1%)
Unknown 7		1/29 (3.4%)		1/66 (1.5%)	1/87 (1.1%)
Unknown 8			1/21 (4.7%)		1/87 (1.1%)
Unknown 9			1/21 (4.7%)		1/87 (1.1%)
Unknown 10			1/21 (4.7%)		1/87 (1.1%)
**Protozoan**					
**Ciliophora**					
Epistylis			1/21 (4.7%)		1/87 (1.1%)

The phylum *ascomycota* was identified in 96.1% (25/26) of preterm infants and 76.9% (10/13) of term babies (*p* = 0.119), while the phylum *basidiomycota* was found in 38.4% (10/26) of preterm infants and 23% (3/13) of term babies (*p* = 0.548).

*Cladosporium* was the predominant genus in both term 38.4% (5/13) and preterm infants 73% (19/26), without statistical differences between them (*p* = 0.08). Among preterm infantas, it was more frequent in the respiratory distress group (84.4%, 11 of 13 *vs*. 61.5%, 8 of 13; *p* = 0.378). *Candida* was the second genus in frequency being identified in 15.4%, 2 of 13 of term infants and in 46.1%, 12 of 26 of preterm newborns (*p* = 0.12). The most frequently identified species was *Candida sake*, all in 10 preterm newborns, five in each group. There was a case of *Candida parapsilosis* in a preterm newborn with respiratory distress syndrome. *P*. *jirovecii* was only identified among preterm infants with a frequency of 26.7% (7 of 26) with no significant differences between babies with and without RDS.

Overall, *Malassezia* was the predominant genus in the *Basidiomycota* phylum, but only present among preterm infants (3 out 26; 11.5%). There was a case of *Cryptococcus neoformans* in a preterm newborn with respiratory distress syndrome. Ten unknown fungi were detected from eight patients and a protozoan of the genus *Epistylis* in a term newborn.

**Fig 1:**
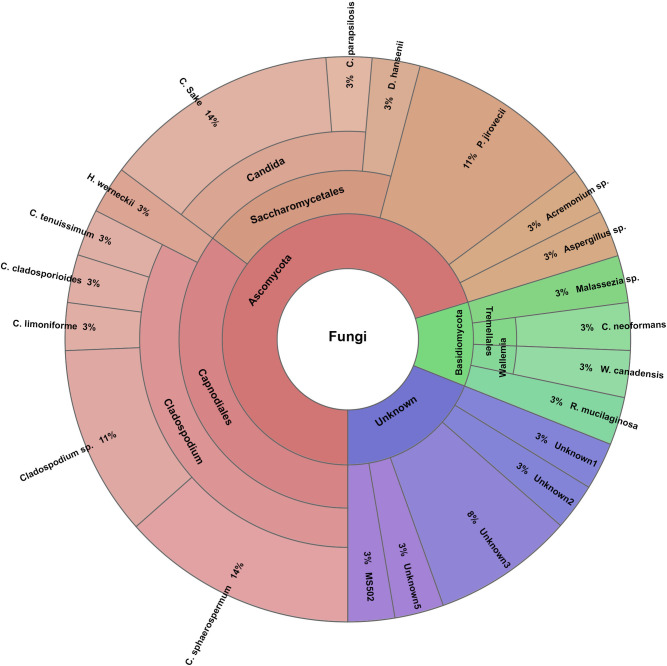
Distribution of the airway mycobiota among preterm infants with respiratory distress syndrome.

**Fig 2:**
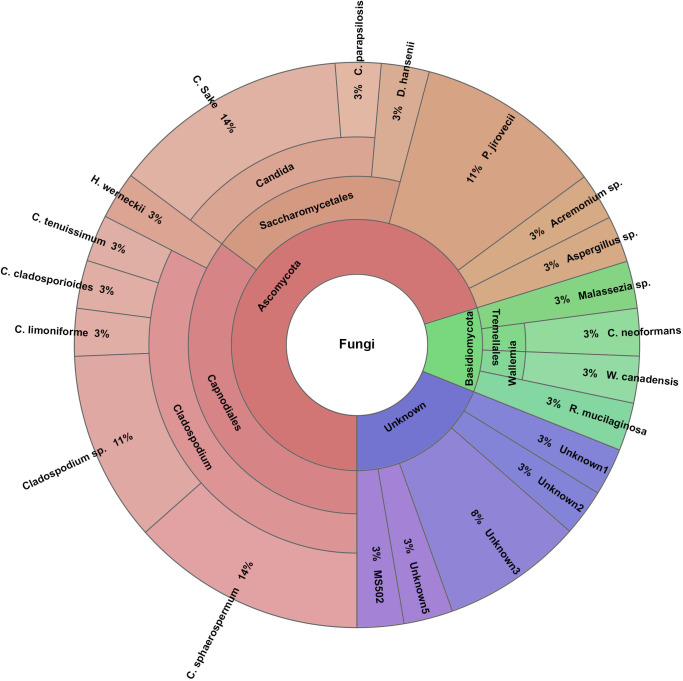
Distribution of the airway mycobiota among preterm infants without respiratory distress syndrome.

The airway mycobiota in preterm infants with and without RDS are shown in Table 2 and Figs 1 and 2.

## Discussion

This study describes, for the first time, the presence of multiple fungal genus in the airways of newborns and compares between term and premature infants at birth. The fungi of the *Ascomycota* phylum as a whole represent the main taxa present in preterm newborns. They are predominant in both newborns with and without RDS. Compared with this, the *Basidiomycota* phylum, although as a whole less frequent in both groups of infants than *Ascomycota*, was more frequent among newborns without RDS.

Curiously, *C*. *sake* and *P*. *jirovecii* were only found in preterm newborns. *C*. *sake* has been associated with early caries in children and occasionally with severe infections including fungal endocarditis, peritonitis, and candidaemia [14, 15]. *P*. *jirovecii* has been associated with RDS and bronchopulmonary dysplasia (BPD) in preterm infants [10, 16]. The lack of association in this study could be related to the sample size. However, the potential relationship between *C*. *sake* or *P*. *jirovecii* and the risk of prematurity deserves further studies.

Until now, the possible relationship between the fungal microbiota of the airways and respiratory disease in preterm infants has not been studied. However, different studies have evaluated the relationship between the bacterial microbiota and respiratory disease in preterm infants. Stressmann *et al*. speculated that the presence in the airways of eight intubated preterm infants of *Staphylococcus aureus*, *Enterobacter sp*, *Moraxella catarrhalis*, *Pseudomona aeruginosa* or *Streptococcus sp* could be related to the subsequent development of respiratory complications in premature newborns [17]. Mourani *et al*. found early bacterial colonization after the first 72 hours of life in the airways of ten intubated preterm infants, most frequently *Staphylococus spp* or *Ureaplasma spp*, which could be involved in the subsequent development of respiratory complications [18]. In fact, several microorganisms have been involved in the development of BPD such as *Chlamydia*, *Ureaplasma*, *Mycoplasma*, *citomegalovirus*, and *adenovirus*, but not with RDS [19–26].

Using Next-generation sequencing-based detection in the gastric fluid of neonates with respiratory distress, *Ureoplasma* was detected in two of the ten cases and *Streotococcus* in three of the seven cases, but only an association with chorioamnionitis could be established for *Uroplasma* [27].

Recently, in a study carried out in Mexico in newborns with respiratory distress, the presence of *Chlamydia* DNA was identified in 34.1% of the bronchial lavages studied, while the prevalence of *C*. *trachomatis* infection in pregnant Mexican women is 6.73% [28, 29].

Several studies observed findings suggesting that a diverse microbiota is related to healthier states [30–32]. However, in our study, we found a greater number of fungal species in preterm infants with RDS. Fungal infection has been hypothesized to contribute to creating an inflammatory state that can interfere with bacterial colonization or increase its alveolar clearance. In this sense, a metagenomic study of the respiratory track microbiota of patients with idiopathic interstitial pneumonia showed a clearly negative relationship between the presence of *P*. *jirovecii* and the bacterial microbiota, suggesting an antagonistic relationship [33]. In the same way, a higher frequency of fungi in preterm infants could induce a decrease in bacterial community and thus play a role in RDS.

There are several limitations to our study. First, due to the small sample size, we cannot conclude significant differences in the fungal microbiota in preterm infants with and without RDS. Furthermore, we analyzed nasopharyngeal aspirate samples so that we could not know the lower airways in infants who did not require mechanical ventilation.

In conclusion, this is the first report to describe the fungal microbiota of the airways in term and preterm infants. Although no differences have been observed, the number of cases analyzed has been small to obtain conclusive results. Therefore, more studies are needed to examine and understand the role that the fungal microbiota of the airways plays in neonatal respiratory pathology.

On the other hand, the presence of *C*. *sake* and *P*. *jirovecii* only in preterm newborns, but not in term infants, suggests a potential relationship with the risk of prematurity. This finding might have clinical relevance that warrants further investigation.

## References

[1] Blencowe H, Cousens S, Oestergaard MZ, et al. National, regional, and worldwide estimates of preterm birth rates in the year 2010 with time trends since 1990 for selected countries: a systematic analysis and implications. *Lancet* 2012; 379 (9832): 2162–72. doi: 10.1016/S0140-6736(12)60820-4 22682464

[2] Kinney MV, Lawn JE, Howson CP, Belizan J. 15 Million preterm births annually: what has changed this year? *Reprod Health* 2012; 9, 28: doi: 10.1186/1742-4755-9-28 23148557 PMC3546852

[3] Liu L, Oza S, Hogan D, et al. Global, regional, and national causes of under-5 mortality in 2000–15: an updated systematic analysis with implications for the Sustainable Development Goals. *Lancet* 2016; 388(10063): 3027–3035. doi: 10.1016/S0140-6736(16)31593-8 27839855 PMC5161777

[4] Goedicke-Fritz S, Härtel C, Krasteva-Christ G, Kopp MV, Meyer S, Zemlin M. Preterm Birth Affects the Risk of Developing Immune-Mediated Diseases. *Front Immunol* 2017; 8: 1266. doi: 10.3389/fimmu.2017.01266 29062316 PMC5640887

[5] Hogden L, Munger K, Duffek S. Neonatal Respiratory Distress. *S D Med*. 2021;74: 28–35. 33691054

[6] Sweet DG, Carnielli V, Greisen G, et al. European Consensus Guidelines on the Management of Respiratory Distress Syndrome—2019 Update. *Neonatology* 2019; 115: 432–450. doi: 10.1159/000499361 30974433 PMC6604659

[7] Rubarth LB, Quinn J. Respiratory Development and Respiratory Distress Syndrome. *Neonatal Netw* 2015;34: 231–8. doi: 10.1891/0730-0832.34.4.231 26802638

[8] Warner B, Hamvas A. Lungs, microbes and the developing neonate. *Neonatology* 2015, 107, 337–343. doi: 10.1159/000381124 26044101 PMC4465447

[9] Zhang DW, Lu JL, Dong BY, Fang MY, Xiong X, Qin XJ, et al. Gut microbiota and its metabolic products in acute respiratory distress syndrome. *Front Immunol* 2024;15: 1330021. doi: 10.3389/fimmu.2024.1330021 38433840 PMC10904571

[10] Rojas P, Friaza V, García E, de la Horra C, Vargas SL, Calderón, EJ, et al. Early Acquisition of Pneumocystis jirovecii Colonization and Potential Association with Respiratory Distress Syndrome in Preterm Newborn Infants. *Clin Infect Dis* 2017; 65: 976–981. doi: 10.1093/cid/cix454 28520902

[11] Beser J, Hagblom P, Fernandez V. Frequent in vitro recombination in internal transcribed spacers 1 and 2 during genotyping of Pneumocystis jirovecii. *J Clin Microbiol* 2007;45: 881–6. doi: 10.1128/JCM.02245-06 17202274 PMC1829102

[12] Nahimana A, Francioli P, Blanc DS, Bille J, Wakefield AE, Hauser PM. Determination of the copy number of the nuclear rDNA and beta-tubulin genes of Pneumocystis carinii f. sp. hominis using PCR multicompetitors. *J Eukaryot Microbiol* 2000; 47: 368–72. doi: 10.1111/j.1550-7408.2000.tb00062.x 11140450

[13] Montes-Cano MA, de la Horra C, Martin-Juan J, Varela JM, Torronteras R, Respaldiza N, et al. Pneumocystis jiroveci genotypes in the Spanish population. *Clin Infect Dis* 2004; 39: 123–8. doi: 10.1086/421778 15206063

[14] Baraniya D, Chen T, Nahar A, et al. Supragingival mycobiome and inter-kingdom interactions in dental caries. *J Oral Microb*iol 2020;12: 1729305. doi: 10.1080/20002297.2020.1729305 32158514 PMC7048226

[15] Juneja D, Borah AK, Nasa P, Singh O, Javeri Y, Dang R. Candida sake candidaemia in non-neutropenic critically ill patients: a case series. *Crit Care Resusc* 2011; 13: 187–91. 21880007

[16] Szydłowicz M, Królak-Olejnik B, Vargas SL, et al. Pneumocystis jirovecii colonization in preterm newborns with respiratory distress syndrome. *J Infect Dis* 2022; 225: 1807–1810. doi: 10.1093/infdis/jiab209 33857302 PMC9113508

[17] Stressmann FA, Connett GJ. Goss K, et al. The use of culture-independent tools to characterize bacteria in endo-tracheal aspirates from pre-term infants at risk of bronchopulmonary dysplasia. *J Perinat Med* 2010; 38: 333–7. doi: 10.1515/jpm.2010.026 20121490

[18] Mourani PM, Harris JK, Sontag MK, Robertson CE, Abman SH. Molecular identification of bacteria in tracheal aspirate fluid from mechanically ventilated preterm infants. *PLoS One* 2011; 6: e25959. doi: 10.1371/journal.pone.0025959 22016793 PMC3189942

[19] Castro-Alcaraz S, Greenberg EM, Bateman DA, Regan JA. Patterns of colonization with Ureaplasma urealyticum during neonatal intensive care unit hospitalizations of very low birth weight infants and the development of chronic lung disease. *Pediatrics* 2002; 110: e45. doi: 10.1542/peds.110.4.e45 12359818

[20] Cassell GH, Waites KB, Watson HL, Crouse DT, Harasawa R. Ureaplasma urealyticum intrauterine infection: role in prematurity and disease in newborns. *Clin Microb Rev* 1993; 6: 69–87. doi: 10.1128/CMR.6.1.69 8457981 PMC358267

[21] Hannaford K. Todd DA, Jeffery H, John E, Blyth K, Gilbert GL. Role of Ureaplasma urealyticum in lung disease of prematurity. *Arch Dis Child Fetal Neonatal Ed* 1999; 81: F162–7. doi: 10.1136/fn.81.3.f162 10525015 PMC1721014

[22] López-Hurtado M, Zamora-Ruiz A, Flores-Medina S, Guerra-Infante FM. Prevalence of Chlamydia trachomatis in newborn infants with respiratory problems. *Rev Latinoam Microbiol* 1999; 41: 267–72. 10932768

[23] Couroucli XI, Welty SE, Ramsay PL, et al. Detection of microorganisms in the tracheal aspirates of preterm infants by polymerase chain reaction: association of adenovirus infection with bronchopulmonary dysplasia. *Pediatr Res* 2000; 47: 225–32. doi: 10.1203/00006450-200002000-00013 10674351

[24] Kelly MS, Benjamin DK, Puopolo KM, et al. Postnatal Cytomegalovirus Infection and the Risk for Bronchopulmonary Dysplasia. *JAMA Pediatr* 2015; 169: e153785. doi: 10.1001/jamapediatrics.2015.3785 26642118 PMC4699399

[25] Viscardi RM, Kallapur SG. Role of ureaplasma respiratory tract colonization in bronchopulmonary dysplasia pathogenesis: current concepts and update. *Clin Perinatol* 2015; 42: 719–38. doi: 10.1016/j.clp.2015.08.003 26593075 PMC4662049

[26] Iles R, Lyon A, Ross P, McIntosh N. Infection with Ureaplasma urealyticum and Mycoplasma hominis and the development of chronic lung disease in preterm infants. *Acta Paediatric* 1996; 85: 482–4. doi: 10.1111/j.1651-2227.1996.tb14067.x 8740310

[27] Okumura T, Horiba K, Tetsuka N, Sato Y, Sugiyama Y, Haruta K, et al. Next-generation sequencing-based detection of Ureaplasma in the gastric fluid of neonates with respiratory distress and chorioamnionitis. J Matern Fetal Neonatal Med. 2023; 36:2207113. doi: 10.1080/14767058.2023.2207113 37150592

[28] González-Fernández MD, Escarcega-Tame MA, López-Hurtado M, Flores-Salazar VR, Escobedo-Guerra MR, Giono-Cerezo S, et al. Identification of Chlamydia trachomatis genotypes in newborns with respiratory distress. An Pediatr (Engl Ed). 2023; 98: 436–445. doi: 10.1016/j.anpede.2023.04.010 37169687

[29] López-Hurtado M, García-Romero S, Escobedo-Guerra M, Bustos-López D, Guerra-Infante F. Prevalencia de la infección genital por Chlamydia trachomatis en mujeres que asisten al Instituto Nacional de Perinatología de la Ciudad de México. Rev Chilena Infectol. 2018; 35:371–6. 30534923 10.4067/s0716-10182018000400371

[30] Lal CV, Travers C, Aghai ZH, et al. The Airway Microbiome at Birth. *Sci Rep* 2016, 6, 31023. doi: 10.1038/srep31023 27488092 PMC4973241

[31] Cho I, Blaser MJ. The human microbiome: at the interface of health and disease. *Nat Rev Genet* 2012, 13, 260–70. doi: 10.1038/nrg3182 22411464 PMC3418802

[32] Hilty M, Burke C, Pedro H, et al. Disordered microbial communities in asthmatic airways. *PLoS One* 2010; 5: e8578. doi: 10.1371/journal.pone.0008578 20052417 PMC2798952

[33] Friaza V. de la Horra C, Rodríguez-Domínguez MJ, Cantón R Martín-Juan J, Calderón EJ, Del Campo R. Metagenomic analysis of bronchoalveolar lavage simples from patients with idiopathic interstitial pneumonia and its antagonic relation with Pneumocystis jirovecii colonization. *J Microbiol Methods* 2010; 82: 98–101. doi: 10.1016/j.mimet.2010.03.026 20382190

